# TBC1 domain family member 23 interacts with Ras‐related protein Rab‐11A to promote poor prognosis of non‐small‐cell lung cancer via β1‐integrin

**DOI:** 10.1111/jcmm.16841

**Published:** 2021-08-07

**Authors:** Yao Zhang, Hongbo Su, Muli Wudu, Hongjiu Ren, Yitong Xu, Qingfu Zhang, Jun Jiang, Qiongzi Wang, Xizi Jiang, Bo Zhang, Zongang Liu, Zifang Zou, Xueshan Qiu

**Affiliations:** ^1^ Department of Pathology First Affiliated Hospital College and of Basic Medical Sciences China Medical University Shenyang China; ^2^ Department of Pathology Basic Medical Sciences Xinjiang Medical University Urumqi China; ^3^ Department of Thoracic Surgery The First Hospital of China Medical University Shenyang China; ^4^ Department of Thoracic Surgery Shengjing Hospital of China Medical University Shenyang China

**Keywords:** integrin beta 1, non‐small‐cell lung cancer, prognosis, RAB11A protein, TBC1D23 protein

## Abstract

Non‐small‐cell lung cancer (NSCLC) accounts for approximately 80% of lung cancer cases. TBC1D23, a member of the TBC/RABGAP family, is widely expressed in human tissues; however, its role in NSCLC is currently unknown. Immunohistochemical analysis was conducted on 173 paraffin‐embedded lung tissue sections from patients with NSCLC from 2014 to 2018 at the First Affiliated Hospital of China Medical University. MTT, colony formation assay, cell cycle assay, scratch assay, transwell assay, Western blotting and real‐time PCR were employed on multiple NSCLC cell lines modified to knock down or overexpress TBC1D23/RAB11A. Immunoprecipitation, immunoprecipitation‐mass spectrometry, immunofluorescence and flow cytometry were performed to explore the interaction between TBC1D23 and RAB11A and TBC1D23 involvement in the interaction between RAB11A and β1 integrin in the para‐nucleus. TBC1D23 was correlated with tumour size, differentiation degree, metastasis, TNM stage and poor prognosis. TBC1D23 was involved in the interaction between RAB11A and β1 integrin in the para‐nucleus, thus activating the β1 integrin/FAK/ERK signalling pathway to promote NSCLC. Furthermore, TBC1D23 promoted NSCLC progression by inducing cell proliferation, migration and invasion. This study indicated the relationship between TBC1D23 expression and the adverse clinicopathological characteristics of patients with NSCLC, suggesting that TBC1D23 may be an important target for NSCLC treatment.

## 1 INTRODUCTION

Lung cancer is a malignant tumour with the highest morbidity and mortality in the world, posing a serious threat to human health and demonstrating an annually increasing trend.^1^ According to the 2018 global cancer statistics, lung cancer patients accounted for 11.6% of the total number of malignant tumours and 18.4% of all malignant tumour‐related deaths. Among these, NSCLC accounts for approximately 80% of cases.^2^, ^3^, ^4^ The five‐year survival rate for NSCLC is no more than 15%.^5^, ^6^

TBC1D23, a member of the TBC/RABGAP family, is widely expressed in human tissues and cell lines. The TBC/RABGAP family of proteins has a TBC (Tre2‐Bub2‐Cdc16) domain^7^ that regulates RAB GTPase activation (RABGAP).^8^ Previous research has demonstrated the important role played by TBC1D23 in intracellular substance transport.^9^, ^10^ TBC1D23 also affects neuron growth, brain development and autoimmunity.^11^, ^12^ However, unlike other members of the TBC family, the TBC domain of TBC1D23 is catalytically inactive and lacks the catalytic Arg and Gln residues needed for RABGAP function.^13^ Marin‐Valencia et al. reported that TBC1D23 had no RABGAP function, but this did not preclude interactions between TBC1D23 and the RAB family.^14^

RAB11A, a member of the RAB small GTPase family, is widely expressed in mammals.^15^ RAB11A is located in recycling endosomes, the trans‐Golgi network and the plasma membrane.^16^ It plays an important role in ligand and receptor recycling, cytokinesis, cell secretion and intracellular signal transduction.^17^, ^18^, ^19^ Recent studies have demonstrated that RAB11A can affect tumour development through regulation of β1‐integrin circulation.^20^, ^21^ β1‐integrin, a crucial transmembrane receptor,^22^ promotes disease development via multiple signalling pathways.^23^ The FAK pathway is one of the most important pathways in cancer development.^24^ The MAPK pathway is a common downstream signalling pathway of FAK, which is activated in turn by a tertiary kinase cascade.^25^, ^26^ The β1‐integrin/FAK/MAPK signalling is an essential mechanism of tumorigenesis and development, involved in inducing epithelial‐mesenchymal transition, promoting tumour cell proliferation, inhibiting tumour cell apoptosis and promoting tumour angiogenesis. Therefore, many anti‐tumour drugs have been developed to inhibit this pathway, with the goal of delaying tumour progression.^27^, ^28^

The current study aimed to identify the relationship between TBC1D23 expression and NSCLC and to determine whether TBC1D23 promotes the proliferation, migration and invasion of NSCLC cells. Furthermore, the interaction between TBC1D23 and RAB11A was investigated to determine its influence on the β1 integrin/FAK/ERK signalling pathway.

## 2 MATERIALS AND METHODS

### 2.1 Patients and specimens

This study included 173 lung tissue samples selected after surgery from patients diagnosed with NSCLC from 2014 to 2018 at the First Affiliated Hospital of China Medical University. Patients did not receive radiotherapy or chemotherapy before surgery. Written informed consent was obtained from each patient, and the study was approved by the Medical Research Ethics Committee of China Medical University.

### 2.2 Immunohistochemistry (IHC)

The tissues of patients with NSCLC were fixed with 10% formalin, embedded in paraffin and cut into 4 µm‐thick slices. After de‐paraffinization, antigen retrieval was performed using 0.01% citrate buffer (Fuzhou Maixin Biotech Co., Ltd., Fujian) for 3 min. After respective treatments with hydrogen peroxide and 5% goat serum at 37°C for 1 h, the sections were incubated overnight at 4°C with the primary antibodies anti‐TBC1D23 (1:200; ProteinTech Group) and anti‐β1 integrin (1:200; ProteinTech Group). The Elivision Super HRP (Mouse/Rabbit) IHC Kit (Fuzhou Maixin Biotech Co., Ltd.) was used for 20 min at 37°C, followed by treatment with 3,3'‐diaminobenzidine for 2 min. Finally, cell nuclei in the sections were stained with haematoxylin (Fuzhou Maixin Biotech Co., Ltd.).

We scored the specimens according to IHC staining results: 0 (no staining), 1 (weak staining), 2 (medium staining) and 3 (strong staining). We further scored the specimens according to IHC staining area: 1 (1%–25%); 2 (26%–50%); 3 (51%–75%); and 4 (76%–100%). The highest score of all slices was 12 points, while the lowest score was 0 points. We regarded scores ≥6 as positive for staining, whereas scores <6 were considered negative for staining.

### 2.3 Western blotting

We used lysis buffer and phenylmethylsulfonyl fluoride (PMSF) to lyse cells and tissues and extract total proteins. The total protein was separated by 10% gel SDS‐PAGE and transferred to polyvinylidene difluoride (PVDF) membranes (Millipore). The membranes were incubated overnight at 4°C with the following primary antibodies: anti‐TBC1D23 (1:1000; #17002‐1‐AP; Proteintech Group), anti‐myc (1:1000; #HT101‐01; TransGen Biotech), anti‐RAB11A (1:1000; 20229–1‐P; Proteintech Group) (1:200; #sc166523; Santa Cruz Biotechnology), anti‐β1 integrin (1:500; #26918‐1‐AP; Proteintech Group), anti‐cyclin B1 (1:1000; #4138; Cell Signaling Technology Inc.), anti‐cyclin D1 (1:1000; 32922; Cell Signaling Technology Inc.), anti‐CDK2 (1:1000; #2546; Cell Signaling Technology Inc.), anti‐CDK6 (1:1000; #13331; Cell Signaling Technology Inc.), anti‐RhoA (1:1000; #2117; Cell Signaling Technology Inc.), anti‐RhoC (1:1000; #3430; Cell Signaling Technology Inc.), anti‐MMP2 (1:1000; #40994; Cell Signaling Technology Inc.), anti‐FAK (1:1000; #3285; Cell Signaling Technology Inc.), anti‐P‐FAK (1:500; #8556; Cell Signaling Technology Inc.), anti‐MEK (1:1000; #WL03328; Wanleibio, Shenyang, China), anti‐P‐MEK (1:1000; #9154; Cell Signaling Technology Inc.), anti‐ERK (1:1000; #4695; Cell Signaling Technology Inc.), anti‐P‐ERK (1:1000; #9101; Cell Signaling Technology Inc.), anti‐P‐JNK (1:1000; #9251; Cell Signaling Technology Inc.), anti‐P‐p38 (1:1000; #9211; Cell Signaling Technology Inc.), anti‐β‐actin (1:10000; #20536‐1‐AP; Proteintech Group) and anti‐GAPDH (1:10000; #10494; Proteintech Group). Membranes were then incubated at 37°C for 1 h with HRP‐conjugated anti‐mouse/rabbit IgG. The results were observed with enhanced chemiluminescence (Thermo Fisher Scientific). The β‐actin or GAPDH content was used as an internal control. Each experiment was repeated in triplicate.

### 2.4 Real‐time PCR

SYBR Green PCR Master Mix was used to extract mRNA from cells and tissues under the following conditions: 95°C for 30 s, 45 cycles at 95°C for 5 s and 60°C for 30 s. The 7900HT Fast Real‐Time PCR System (Applied Biosystems) was used for analysis. The β‐actin content in total mRNA was used as a reference. The following primer sequences were used TBC1D23 forward: 5'‐ATGTTTCCCAGTCATCTGTTGGTTACT‐3' and TC1D23 reverse: 5'‐AACTTTCATGATCTGCTGTTTTATGGC‐3'. β1‐integrin forward: 5'‐CAAGAGAGCTGAAGACTATCCCA‐3', β1‐integrin reverse: 5'‐TGAAGTCCGAAGTAATCCTCCT‐3'. β‐actin forward: 5'‐ATAGCAGCCTGGATAGCAAGTAC‐3', β‐actin reverse: 5'‐CACCTTCTACAATGAGCTGCGTGTG‐3'. Each experiment was repeated in triplicate.

### 2.5 Immunofluorescence

Cells were cultured in a 24‐well plate for 24 h. Cells were then immobilized with 2% paraformaldehyde for 20 min, followed by incubation in 5% BSA for 2 h at 37°C. Cells were incubated overnight at 4°C with primary antibodies anti‐TBC1D23 (1:100; #17002‐1‐AP; Proteintech Group), anti‐RAB11A (1:50; #20229‐1‐P; Proteintech Group) and anti‐β1 integrin (1:50; #26918‐1‐AP; Proteintech Group), followed by incubation with FITC/TRITC‐conjugated secondary antibody for 2 h at 37°C. Finally, cell nuclei were stained using 4',6‐diamidino‐2‐phenylindole (DAPI). Cells were observed using a laser confocal microscope. Each experiment was repeated in triplicate.

### 2.6 Cell culture and treatment

Human bronchial epithelial (HBE) cells were obtained from ATCC and cultured in Dulbecco's modified Eagle's medium (DMEM) (Gibco). NSCLC cell lines including H1299, A549, H460, H661, SK‐MES‐1, H226 and H292 were obtained from the Cell Bank of the Chinese Academy of Sciences (Shanghai). SK‐MES‐1 cells were cultured in MEM (Gibco), while the other cell lines were cultured in RPMI 1640 medium (Gibco). 10% foetal bovine serum (FBS) was added to all media, and cells were cultured at 37°C under an atmosphere containing 5% carbon dioxide.

Transfection was performed using Lipofectamine 3000 Reagent (Invitrogen), according to the manufacturer's instructions. TBC1D23‐specific siRNA and RAB11A‐specific siRNA were used to induce TBC1D23 and RAB11A knockdown, respectively; pcDNA3.0 (donated by Massimo Broggini from Istituto di Ricerche Farmacologiche, Ranica, Italy) and pcDNA3.0‐TBC1D23 plasmids were used for controls and to induce TBC1D23 overexpression, respectively.

After 48 h, a nocodazole (NZ) washout system was established using 10 µM NZ (MedChemExpress, Monmouth Junction). Cells were cultured in serum‐free medium 12 h before dosing. The initial time point was set as 4 h after dosing.

### 2.7 Cell proliferation and colony formation

To measure cell proliferation by MTT assay, cells were inoculated into a 96‐well plate at 3000 cells/100 µl 24 h after transfection and cultured for 5 consecutive days. MTT solution (10 µl/well) was added at the same time every day, and 100 µl DMSO was added after 4 h of dark culture. Finally, the wells were read by a microplate reader at a wavelength of 490 nm. Each experiment was repeated in triplicate.

To measure colony formation, cells were inoculated into a 6‐well plate at 500 cells/4 ml 24 h after transfection and cultured for 10–15 days. Cells were fixed with Giemsa solution, washed with PBS and stained with haematoxylin. Each experiment was repeated in triplicate.

### 2.8 Cell Cycle

Forty‐eight hours after transfection, cells were fixed with 70% ethanol overnight at 4°C. Propidium iodide in PBS supplemented with RNase A (5 mg/ml) was added to the cells for 30 min in the dark at 37°C. Finally, cell cycle was measured using the FACSCalibur flow cytometer (BD Biosciences). Each experiment was repeated in triplicate.

### 2.9 Cell migration and invasion

Scratch assays were performed with cells 48 h after transfection. Cells were cultured in serum‐free medium for 2 h, then a channel was marked in the cells, and the width of the channel was recorded. The width of the channel was measured after incubation for 24 h and the difference between the widths of the two channels was calculated. Each experiment was repeated in triplicate.

For the invasion assay, cells were inoculated into the upper chamber 24 h after transfection at 100,000 cells/200 µl (SK‐MES‐1 cells), 80,000 cells/200 µl (A549 cells) and 60,000 cells/200 µl (H1299 cells) for 20 h; 600 µl serum‐free medium was added into the lower chamber. The medium concentration in the upper chamber was 2% and that in the lower chamber was 20%. The invasion assay was also conducted with Matrigel, in which 100 µl of Matrigel (1:9 dilution; BD Biosciences) was added to the upper chamber a day prior to the experiment. The remaining steps were conducted in the same manner as described in the colony formation assay. Each experiment was repeated in triplicate.

### 2.10 Co‐Immunoprecipitation (Co‐IP) and IP‐mass spectrometry

Immunoprecipitation was conducted in the same manner as described for Western blotting. Proteins of the experimental group were incubated with anti‐TBC1D23 (#17002‐1‐AP; Proteintech Group), anti‐myc (#HT101‐01; TransGen Biotech) or anti‐RAB11A (#sc166523; Santa Cruz Biotechnology) antibodies and protein A+G Agarose beads. Proteins from the control group were incubated with anti‐IgG rabbit/mouse antibodies and protein A+G agarose beads. All proteins were incubated overnight at 4°C. After dissociation from the beads, the denatured immunocomplexes were separated by 10% SDS‐PAGE and stained with Coomassie Blue. Finally, they were sent to Shanghai Applied Protein Technology for mass spectrometry analysis. Each experiment was repeated in triplicate.

### 2.11 Flow cytometry

Cells were digested with trypsin, washed with PBS and collected. Cells were then incubated with FITC‐conjugated antibody to mouse β1 integrin for 30 min at 4°C. Cells were fixed with 4% paraformaldehyde for 20 min at 4°C. Finally, cells were analysed using the FACSCalibur flow cytometer (BD Biosciences). Each experiment was repeated in triplicate.

### 2.12 Statistical Analysis

Chi‐square tests were used to compare immunohistochemistry scoring results using SPSS v17.0 software (SPSS Inc.,) for statistical analysis. Two‐tailed *t* tests were used to compare differences between experimental and control groups using ImageJ software (National Institutes of Health, Bethesda) for statistical analysis. *p* values <0.05 were considered statistically significant.

## 3 RESULTS

### 3.1 TBC1D23 is highly expressed in NSCLC and correlated with poor prognosis

**FIGURE 1:**
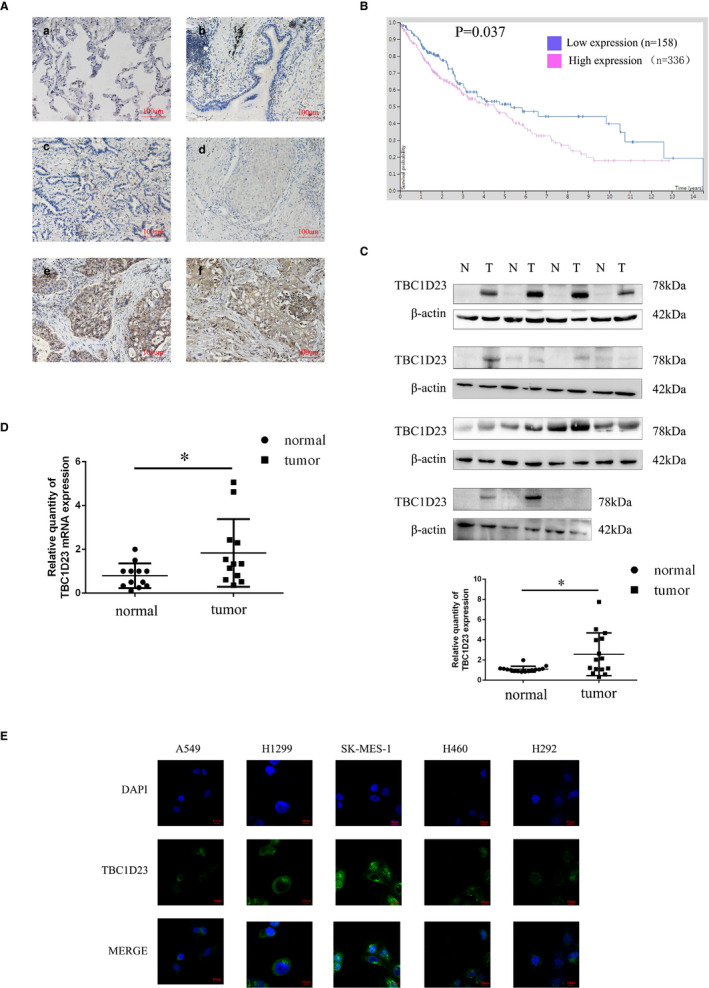
TBC1D23 expression in NSCLC. (A) Immunohistochemistry showing TBC1D23 expression in different tissues, including (A) alveoli; (B) bronchial tubes; (C) highly differentiated adenocarcinoma; (D) poorly differentiated adenocarcinoma; (E) highly differentiated squamous cell carcinoma; and (F) poorly differentiated squamous cell carcinoma. Magnification ×200. (B) Data from the Human Protein Atlas database on the survival of NSCLC patients with high or low TBC1D23 expression levels. (C) Western blot showing higher TBC1D23 expression in NSCLC tissues than in normal tissues for 15 tissue pairs. N: normal tissue(1.097 ± 0.2898), T: NSCLC tissue (2.565 ± 2.122) (D) Real‐time PCR showing higher TBC1D23 mRNA expression in NSCLC tissues than in normal tissues for 12 tissue pairs. N: normal tissue(0.7930 ± 0.5639), T: NSCLC tissue. (1.838 ± 1.547) (E) Immunofluorescence assay for A549, H1299, SK‐Ms‐1, H460 and H292 cells showing TBC1D23 localized in the cytoplasm. Magnification ×1000. *p < 0.05; **p < 0.01; ***p < 0.001. Data are presented as means ± SD (*n* = 3)

IHC revealed positive staining of tumour nests and negative staining of alveolar and bronchial epithelial cells (Figure 1A). High TBC1D23 expression was correlated with tumour size (*p* < 0.001), differentiation degree (*p* < 0.001), metastasis (*p* = 0.027) and TNM stage (*p* < 0.001). TBC1D23 expression was not correlated with sex (*p* = 0.237), age (*p* = 0.920) or tumour type (*p* = 0.416) (Table S1). According to survival curve analysis of 494 cases with NSCLC in the Human Protein Atlas database, high TBC1D23 expression was correlated with poor prognosis for NSCLC (Figure 1B), which concurred with our findings.

Next, we analysed 15 pairs of tissue samples (tumour and normal) using Western blotting, revealing higher TBC1D23 expression in tumour tissues than in normal tissues for 12/15 pairs (*p* = 0.0147) (Figure 1C). Real‐time PCR analysis demonstrated higher TBC1D23 RNA expression in tumour tissues than in normal tissues for 10/15 pairs (*p* = 0.0195), which suggested that TBC1D23 had a higher transcription level in NSCLC (Figure 1D).

Previous studies reported that TBC1D23 was located in the cytoplasm, especially in organelles such as the Golgi apparatus.^7^, ^9^, ^13^ We also detected the localization of TBC1D23. IHC staining indicated that TBC1D23 was located in the cytoplasm (Figure 1A). Immunofluorescence analysis of the NSCLC cell lines A549, H1299, SK‐MES‐1, H460 and H292 confirmed that TBC1D23 was located in the cytoplasm (Figure 1E).

### 3.2 TBC1D23 promotes proliferation of NSCLC

**FIGURE 2:**
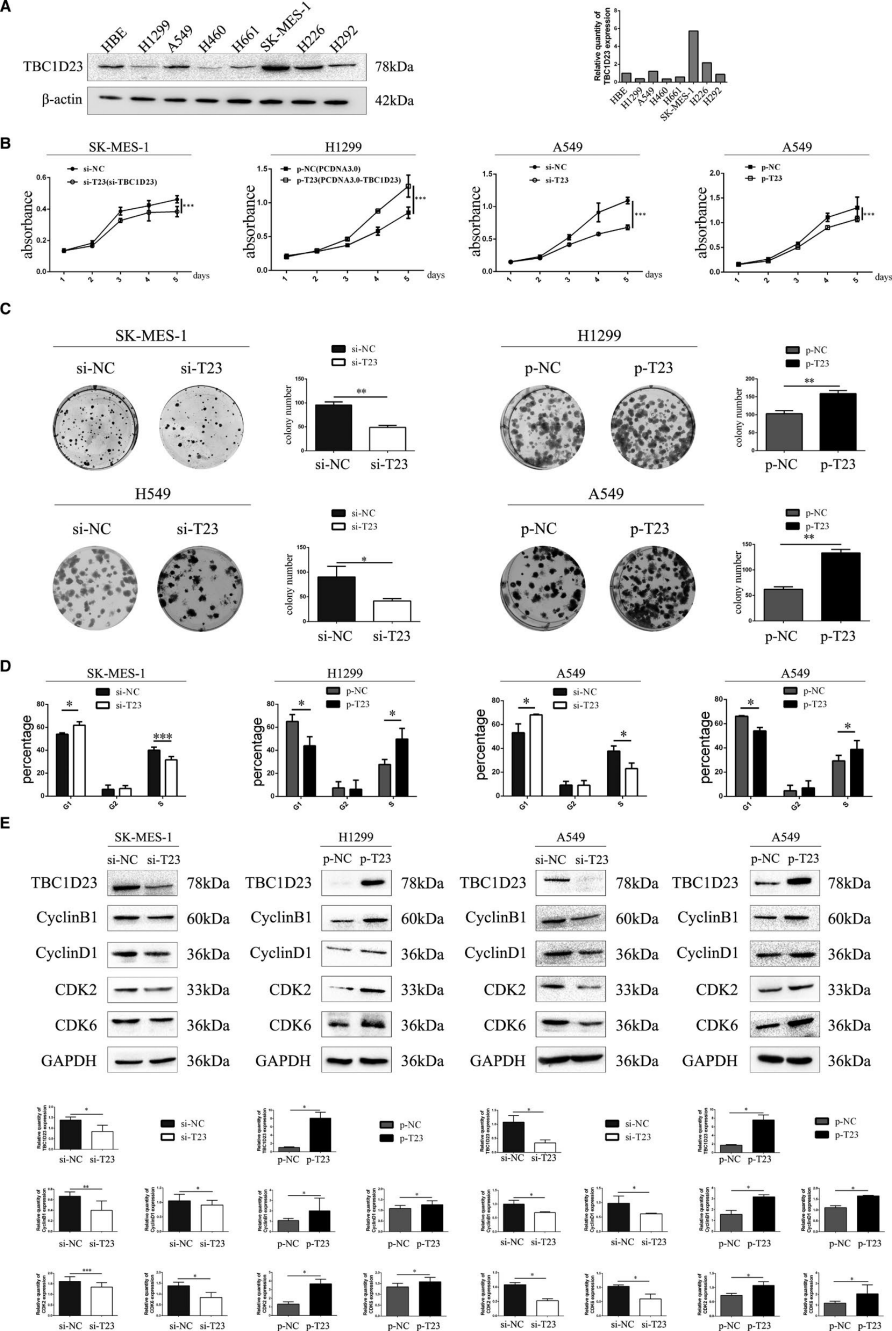
Effects of TBC1D23 on the proliferation of NSCLC cells. (A) Western blot showing TBC1D23 expression in HBE, H1299, A549, H460, H661, SK‐Ms‐1, H226 and H292 cells. (B) MTT assay showing that TBC1D23 promoted the proliferation of NSCLC cells. (C) Colony formation assay showing that TBC1D23 promoted the proliferation of NSCLC cells. (D) Cell cycle assay showing that TBC1D23 promoted the G1/S transition. (E) Western blot showing that TBC1D23 upregulated cyclin B1, cyclin D1, CDK2 and CDK6 to promote the proliferation of NSCLC cells. *p < 0.05; **p < 0.01; ***p < 0.001. Data are presented as means ± SD (*n* = 3)

Western blotting was employed to detect TBC1D23 expression in the NSCLC cell lines H1299, A549, H460, H661, SK‐MES‐1, H226 and H292 and normal HBE cells. The results indicated low TBC1D23 expression in H1299 cells, high expression in SK‐MES‐1 cells and moderate expression in A549 cells (Figure 2A). Therefore, we transfected SK‐MES‐1 cells with NC‐TBC1D23 and si‐TBC1D23 to investigate the effects of TBC1D23 knockdown, transfected H1299 cells with pcDNA3.0 and pcDNA3.0‐TBC1D23 plasmids to investigate the effects of TBC1D23 overexpression and transfected A549 cells with both siRNA and plasmids to confirm the findings.

Since IHC indicated that TBC1D23 was positively correlated with NSCLC tumour size, we investigated the effect of TBC1D23 on the proliferation ability of NSCLC. The results of the MTT and colony formation assays demonstrated that the proliferation and colony‐forming abilities of the TBC1D23 knockdown groups were significantly lower than those of the control groups (Figure 2B,2C). The results of the cell cycle assay revealed that decreased TBC1D23 levels inhibited the G1/S transition (Figure 2D, Figure S3A). Conversely, the cell proliferation and colony formation abilities of the TBC1D23 overexpression groups were significantly higher than those of the control groups (Figure 2B,2C). The results of the cell cycle assays revealed that high TBC1D23 expression level promoted the G1/S transition (Figure 2D).^29^, ^30^ All assays were repeated with transfected A549 cells, and the same results were obtained (Figure 2B–D). Western blot analysis indicated that cyclin B1, cyclin D1, CDK2 and CDK6 were decreased when TBC1D23 was knocked down and increased when TBC1D23 was overexpressed (Figure 2E). Taken together, the results indicated that TBC1D23 promoted NSCLC proliferation.

### 3.3 TBC1D23 promotes migration and invasion of NSCLC

**FIGURE 3:**
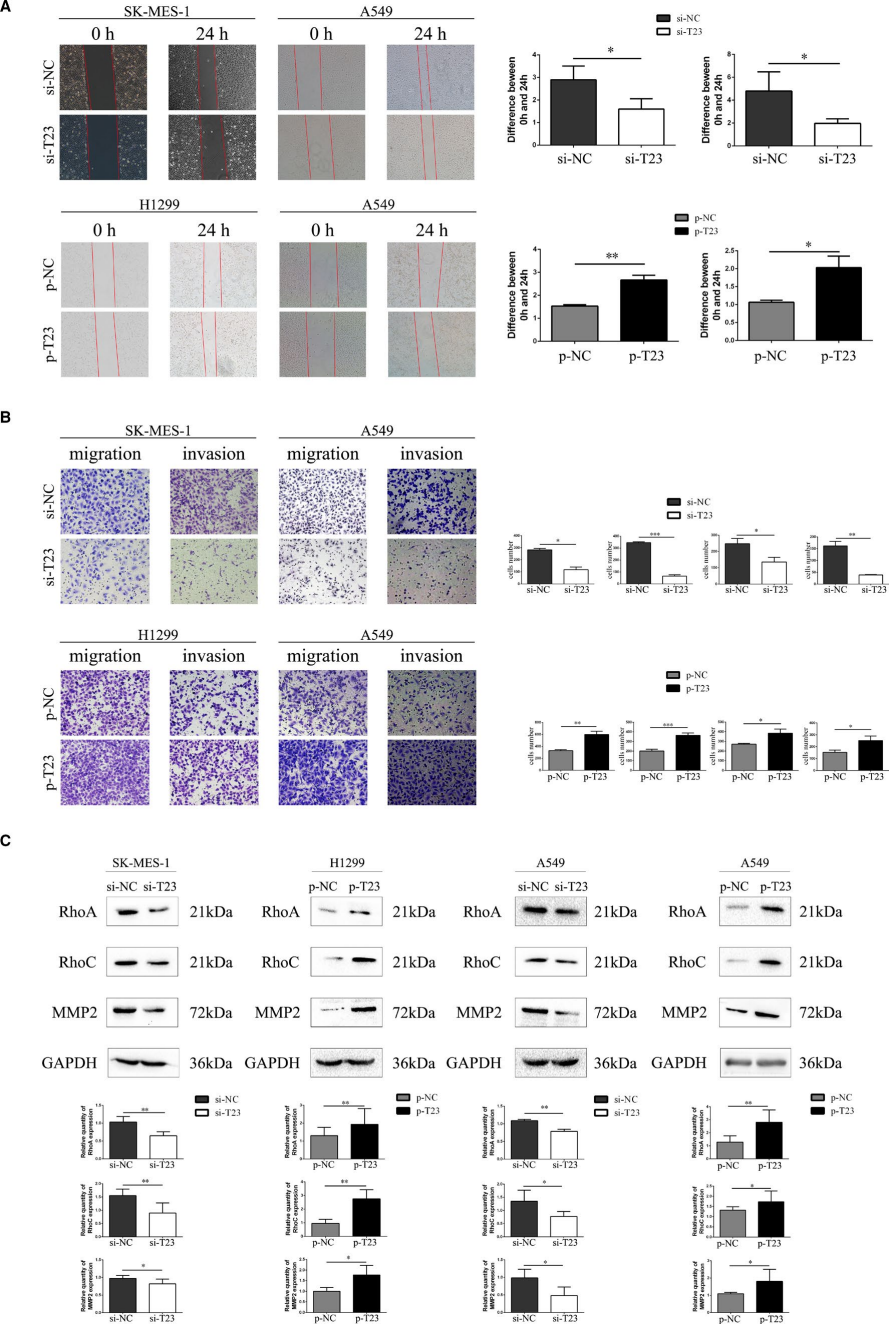
Effects of TBC1D23 on the migration and invasion of NSCLC cells. (A) Scratch assay showing that TBC1D23 promoted the migration of NSCLC cells. Magnification ×100. (B) Transwell assay showing that TBC1D23 promoted the migration and invasion of NSCLC cells. Magnification ×200. (C) Western blot showing that TBC1D23 upregulated RhoA, RhoC and MMP2 to promote the migration and invasion of NSCLC cells. *p < 0.05; **p < 0.01; ***p < 0.001. Data are presented as means ± SD (*n* = 3)

Since IHC indicated that TBC1D23 expression was correlated with lymph node metastasis and TNM stage of NSCLC, we investigated the effect of TBC1D23 on the migration and invasion abilities of NSCLC. First, the results of the scratch and transwell assays demonstrated that the migration ability of the TBC1D23 knockdown SK‐MES‐1 cells was significantly lower than that of the control SK‐MES‐1 cells. The migration ability of the TBC1D23‐overexpressing H1299 cells was greater than that of the control H1299 cells (Figure 3A,3B). Next, the results of the transwell assays with Matrigel revealed that the invasion ability of TBC1D23 knockdown SK‐MES‐1 cells was lower compared with that of control SK‐MES‐1 cells, while the invasion ability of TBC1D23‐overexpressing H1299 cells was greater compared with that of control H1299 cells (Figure 3B). The same results were obtained with transfected A549 cells (Figure 3A,3B). Finally, Western blot analysis indicated that RhoA, RhoC and MMP2 were downregulated when TBC1D23 was knocked down and upregulated when TBC1D23 was overexpressed (Figure 3C, Figure S2). Taken together, the results indicated that TBC1D23 promoted the migration and invasion of NSCLC.

### 3.4 TBC1D23 promotes the biological behaviour of NSCLC by interacting with RAB11A

**FIGURE 4:**
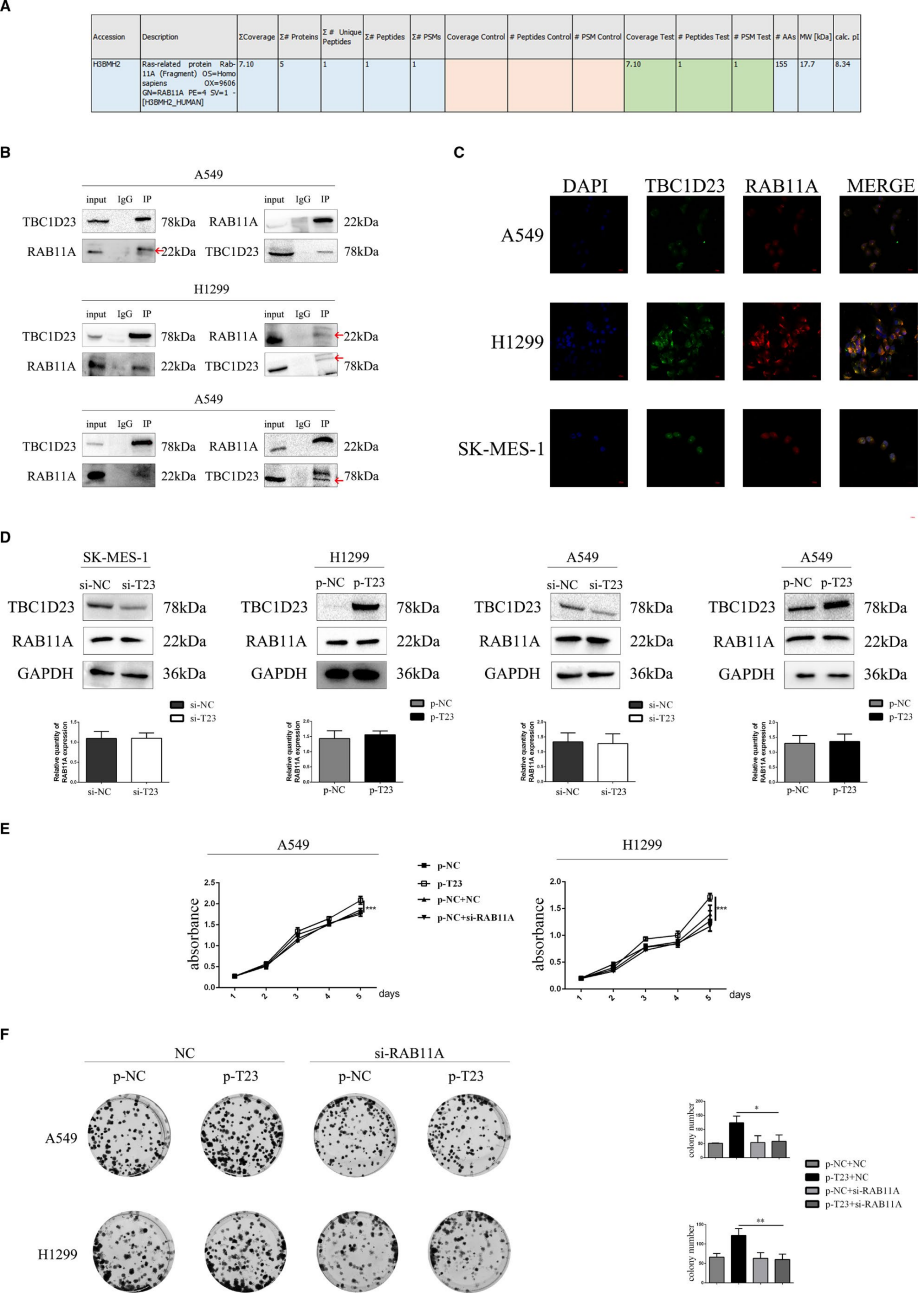
The relationship between TBC1D23 and RAB11A. (A) Mass spectrometry predicted that TBC1D23 could interact with RAB11A. (B) Immunoprecipitation showing that TBC1D23 could interact with RAB11A. (C) Immunofluorescence showing that TBC1D23 and RAB11A were co‐localized in the para‐nucleus. Magnification ×400. (D) Western blot showing that TBC1D23 did not affect RAB11A expression. (E) MTT assay showing that TBC1D23 promoted the proliferation of NSCLC cells by interacting with RAB11A. (F) Colony formation assay showing that TBC1D23 promoted the proliferation of NSCLC cells by interacting with RAB11A. *p < 0.05; **p < 0.01; ***p < 0.001. Data are presented as means ± SD (*n* = 3)

Next, we explored the mechanism responsible for NSCLC promotion by TBC1D23. Firstly, we selected the A549 cell line for immunoprecipitation‐mass spectrometry analysis (IP‐MS) of TBC1D23. We found that TBC1D23 may interact with RAB11A (Figure 4A, Figure S3B,C). Immunoprecipitation in A549 cells revealed that TBC1D23 interacted with RAB11A (Figure 4B). Next, immunofluorescence of A549 cells indicated that TBC1D23 and RAB11A co‐located in the para‐nucleus (Figure 4C), suggesting that TBC1D23 can interact with RAB11A. H1299 and SK‐MES‐1 cells were also analysed by IP and immunofluorescence, and the same results were obtained (Figure 4B,4C).

Does TBC1D23 affect RAB11A expression? RAB11A expression did not change whether TBC1D23 was knocked down or overexpressed in the transfected cells (Figure 4D), indicating that TBC1D23 did not influence RAB11A expression.

**FIGURE 5:**
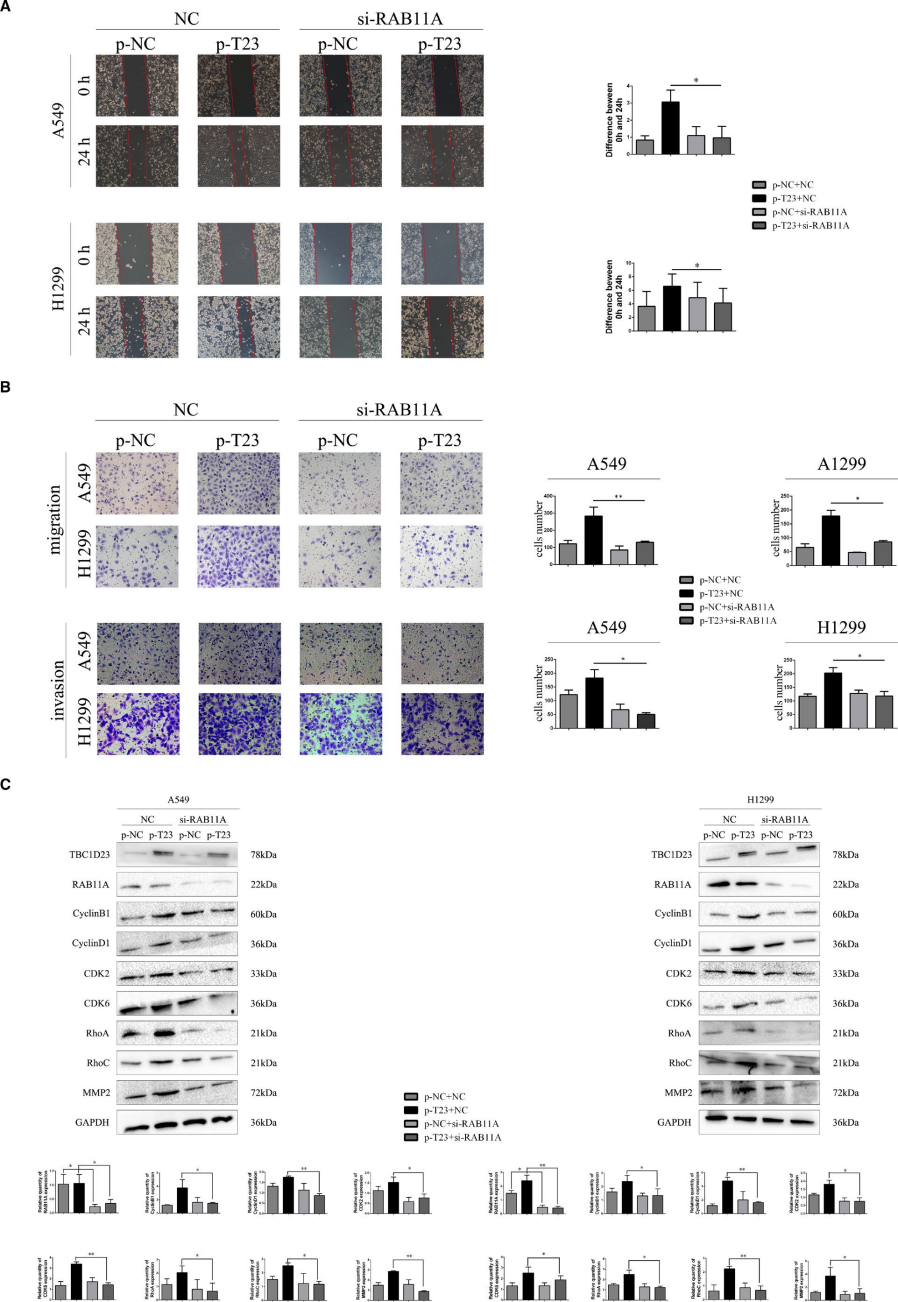
The effect of TBC1D23 and RAB11A interaction on NSCLC cells.(A) Scratch assay showing that TBC1D23 promoted the migration of NSCLC cells by interacting with RAB11A. Magnification ×100. (B) Transwell assay showing that TBC1D23 promoted the migration and invasion of NSCLC cells by interacting with RAB11A. Magnification ×200. (C) Western blot showing that TBC1D23 upregulated cyclin B1, cyclin D1, CDK2 and CDK6 to promote the proliferation of NSCLC cells and upregulated RhoA, RhoC and MMP2 to promote the migration and invasion of NSCLC cells by interacting with RAB11. *p < 0.05; **p < 0.01; ***p < 0.001. Data are presented as means ± SD (*n* = 3)

We then explored whether TBC1D23 promoted NSCLC through its interaction with RAB11A. We employed si‐RAB11A to knock down RAB11A in TBC1D23‐overexpressing A549 cells. The enhancing effect of the TBC1D23 plasmid was restored after RAB11A was knocked down as indicated by the MTT (Figure 4E), colony formation (Figure 4F), scratch (Figure 5A), transwell, and transwell with Matrigel assays (Figure 5B). Western blot analysis revealed that the expression of cyclin B1, cyclin D1, CDK2, CDK6, RhoA, RhoC and MMP2 was restored (Figure 5C). The same results were obtained for TBC1D23‐overexpressing H1299 cells (Figure 4E,F and 5A–C). These results suggested that TBC1D23 promoted NSCLC by interacting with RAB11A.

### 3.5 TBC1D23 interacts with RAB11A to activate the β1 integrin/FAK/ERK pathway

**FIGURE 6:**
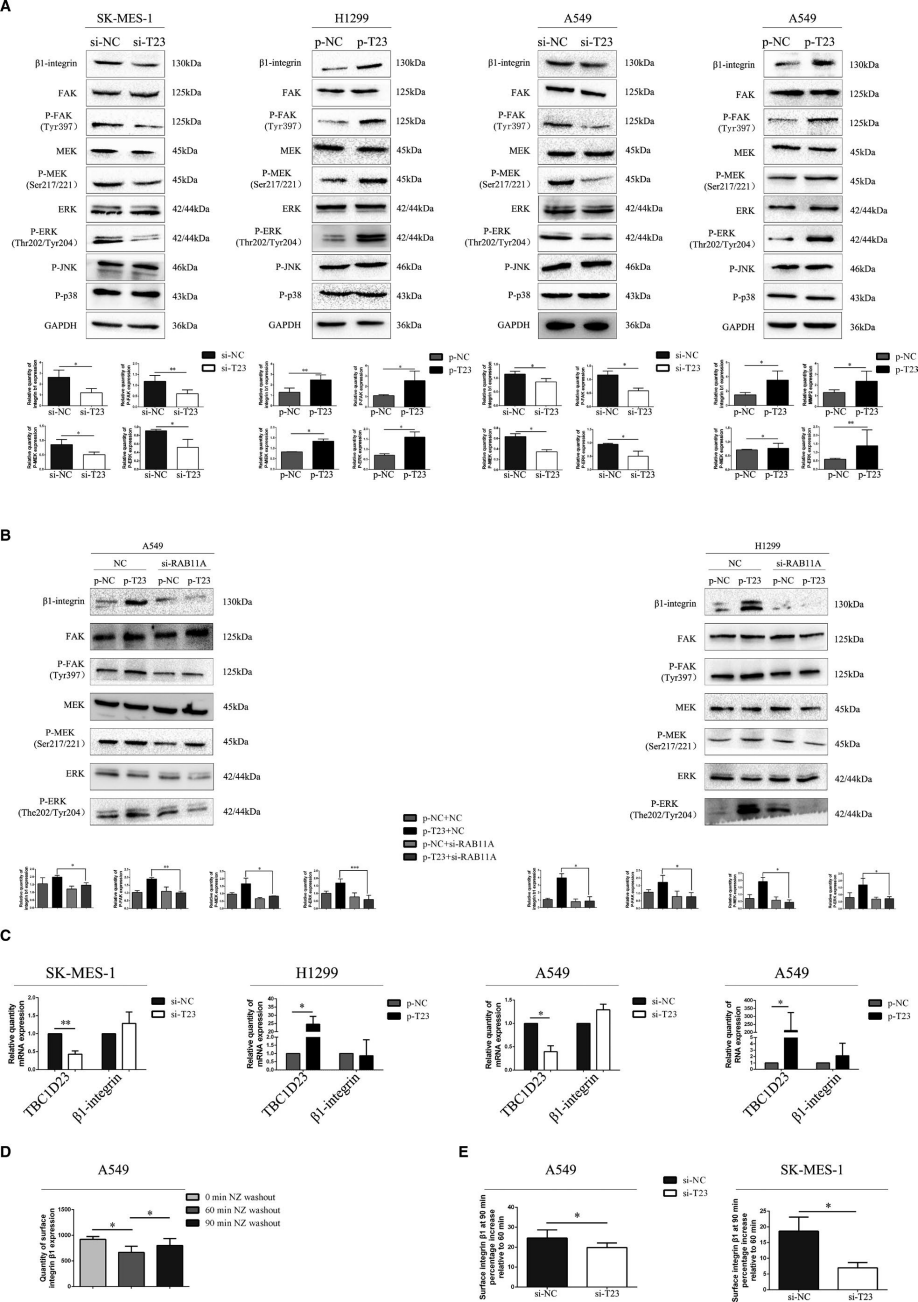
The effect of TBC1D23 on the β1‐integrin/FAK/ERK signalling pathway. (A) Western blot showing that TBC1D23 could activate the β1 integrin/FAK/ERK signalling pathway. (B) Western blot showing that TBC1D23 could activate the β1‐integrin/FAK/ERK signalling pathway by interacting with RAB11A. (C) Real‐time PCR showing that TBC1D23 did not affect β1‐integrin mRNA expression. (D) In A549 cells, β1‐integrin expression on the cell membrane was determined by flow cytometry 0, 60 and 90 min after NZ washout. (E) Flow cytometry showing β1‐integrin expression on the cell membrane in experimental and control groups after NZ washout. The vertical axis of the bar chart shows an increase of 90 min over 60 min. *p < 0.05; **p < 0.01; ***p < 0.001. Data are presented as means ± SD (*n* = 3)

Next, we explored how TBC1D23 interacts with RAB11A to promote NSCLC. Western blot analysis revealed that the expression of β1 integrin, P‐FAK, P‐MEK, and P‐ERK was downregulated in TBC1D23 knockdown SK‐MES‐1 cells compared with the control SK‐MES‐1 cells. However, expression of P‐JNK and P‐p38 was unaltered (Figure 6A). Expression of β1 integrin, P‐FAK, P‐MEK and P‐ERK was upregulated in TBC1D23‐overexpressing H1299 cells compared with control H1299 cells. Again, expression of P‐JNK and P‐p38 was unaltered (Figure 6A). Corresponding results were obtained with A549 cells (Figure 6A). Taken together, the results suggested that TBC1D23 promoted NSCLC by activating the β1 integrin/FAK/ERK pathway.

Next, we knocked down RAB11A in TBC1D23‐overexpressing A549 cells. Expression of β1 integrin, P‐FAK, P‐MEK and P‐ERK was restored. We obtained the same result in TBC1D23‐overexpressing H1299 cells (Figure 6B). These results indicated that TBC1D23 interacted with RAB11A to activate the β1 integrin/FAK/ERK pathway, which promoted the biological behaviour of NSCLC.

Finally, we constructed a TBC1 domain‐deleted splice variant (△TBC1) (Figure S1A). Firstly, we transfected the wild plasmid of TBC1D23 (WT) and △TBC1 into A549 cells for immunoprecipitation analysis and found that without the △TBC1 domain, TBC1D23 could not interact with Rab11A (Figure S1B). Then, we transfected WT and △TBC1 in both A549 and H1299 cells. MTT and colony assays proved that △TBC1 could not promote the proliferation of NSCLC cells (Figure S1C,D). The wound healing, transwell, and transwell with Matrigel assays proved that △TBC1 could not promote the migration and invasion of NSCLC cells (Figure S1E–G). Finally, Western blotting analysis showed that △TBC1 could not promote the expression of CyclinB1, CyclinD1, CDK2, CDK6, RhoA, RHOC, MMP2, β1 integrin, p‐FAK, p‐MEK and p‐ERK in NSCLC cells (Figure S1H). These findings suggested that TBC1D23 interacted with Rab11A through the TBC1 domain, thereby activating the β1 integrin/FAK/ERK signalling pathway, ultimately promoting the proliferation, migration and invasion of NSCLC cells.

### 3.6 TBC1D23 is involved in the interaction between RAB11A and β1 integrin in the para‐nucleus

We further explored the mechanism underlying promotion of NSCLC by TBC1D23. Results of real‐time PCR indicated that β1 integrin mRNA expression was unaltered whether TBC1D23 was knocked down or overexpressed (Figure 6C), indicating that TBC1D23 affected β1 integrin at the post‐transcriptional stage.

According to Nader et al., the microtubule regrowth system after NZ washout can be used to synchronize disassembly of focal adhesions, thus synchronizing β1 integrin to initiate internal circulation.^22^ β1‐Integrin reportedly combines with RAB11A in the para‐nucleus endocytic recycling compartment (PERC) 60 min after NZ washout.^21^ In the current study, IP demonstrated that RAB11A could interact with β1 integrin (Figure S2A).

We validated the method by Nader et al. in A549 cells without transfection and found that maximum β1 integrin content was recovered from the cell membrane 90 min after NZ washout (Figure 6D). Flow cytometry analysis revealed that the β1 integrin content recycled to the cell membrane was significantly lower in the knockdown groups than in the control groups 90 min after NZ washout (Figure 6E).

**FIGURE 7:**
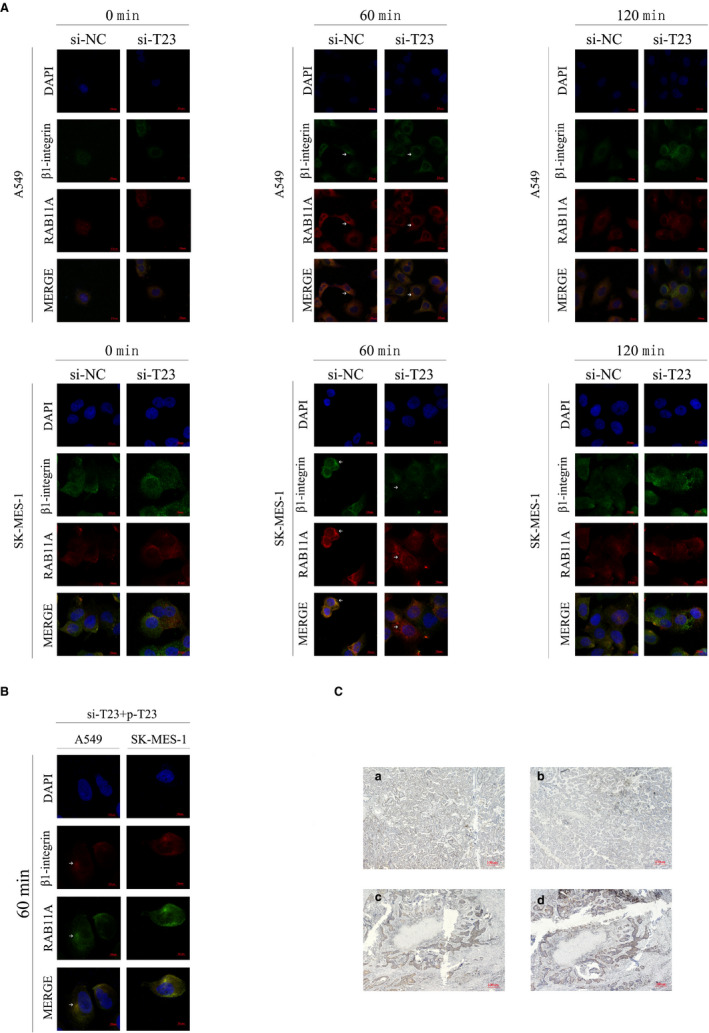
The effect of TBC1D23 on β1‐integrin. (A) Immunofluorescence showing that RAB11A and β1 integrin could not bind in the para‐nucleus when TBC1D23 is knocked down. Magnification ×400. (B)Immunofluorescence showing that RAB11A and β1 integrin were bound in the para‐nucleus after TBC1D23 was overexpressed in cells with TBC1D23 knocked down. Magnification ×400. (C) Immunohistochemistry showing that TBC1D23 was positively correlated with β1‐integrin expression. Magnification ×100 *p < 0.05; **p < 0.01; ***p < 0.001. Data are presented as means ± SD (*n* = 3)

Immunofluorescence analysis then revealed that β1 integrin and RAB11A did not co‐localize in the para‐nucleus in A549 cells with TBC1D23 knockdown 60 min after NZ washout. The same results were obtained for TBC1D23 knockdown SK‐MES‐1 cells (Figure 7A). Next, we overexpressed TBC1D23 in cells with TBC1D23 knockdown and found that RAB11A and β1 integrin were co‐localized in the para‐nucleus at 60 min (Figure 7B). This indicated that TBC1D23 was involved in the interaction between RAB11A and β1 integrin in the para‐nucleus.

Finally, IHC staining of 28 tissue pairs from NSCLC patients indicated that high β1 integrin expression was observed in tissues with high TBC1D23 expression, and the reverse was also true (Figure 7C).

## 4 DISCUSSION

The current study was based on lung tissue samples from 173 patients with NSCLC. TBC1D23 expression in carcinoma nests was significantly higher than that in normal tissues and was associated with poor prognosis. TBC1D23 was shown to promote NSCLC proliferation by upregulating cyclin B1, cyclin D1, CDK2 and CDK6 and promote NSCLC migration and invasion by upregulating RhoA, RhoC and MMP2. Furthermore, our results demonstrated that TBC1D23 promoted the proliferation, migration and invasion of NSCLC by activating the β1 integrin/FAK/ERK pathway. Additionally, TBC1D23 was involved in the interaction between RAB11A and β1‐integrin in the para‐nucleus.

The TBC domain of TBC1D23 lacks the catalytic Arg and Gln residues,^13^ resulting in the loss of RABGAP catalytic function possessed by 55 members of the RAB family, including RAB11A.^14^ However, this does not necessarily mean that TBC1D23 cannot be combined with a member of the RAB family. As Marin‐Valencia et al. suspected, TBC1D23 might be either a binding protein or an effector of RAB, not necessarily a regulator of RAB.^14^ We hypothesized that TBC1D23 may bind to a member of the RAB family, but not catalyse RAB inactivation. Therefore, we selected RAB11A from the results of our IP‐MS analysis to verify our conjecture. According to subsequent results, TBC1D23 simply bound to RAB11A without affecting RAB11A protein expression or regulating RAB11A. Based on the role of TBC1D23 in substance transport mentioned in previous literature, our results suggest that TBC1D23 may change the localization of RAB11A in cells, or help RAB11A to jointly mediate the transportation and alter localization of substances in cells. Immunofluorescence of TBC1D23 and RAB11A revealed that co‐localization of TBC1D23 and RAB11A in cells was not distributed diffusely, but clustered in the para‐nucleus. According to previous studies, a crucial function of RAB11A is to mediate integrin circulation through intracellular recycling back to the cell membrane. Moreover, β1‐integrin binds to ligands on the cell membrane and enters cells through endocytosis.^21^ After screening early endosomes, some β1‐integrin undergoes lysosomal degradation by late endosomes.^31^, ^32^ When separated from the ligand, β1‐integrin can be recycled in two ways. One is a short circulation pathway mediated by RAB4, in which β1 integrin returns directly from the early endosome to the plasma membrane, and the other is a long recycling pathway mediated by RAB11A, in which β1 integrin is returned to the cell membrane through PERC with the assistance of ARF6.^33^ When more β1 integrin is recycled to the cell membrane through PERC, less β1‐integrin is degraded and the overall amount of β1 integrin increases. Therefore, TBC1D23 may form a complex with RAB11A in the PERC. After dissociation from the ligand, β1‐integrin is transported to the PERC. Under joint action of the TBC1D23‐RAB11A complex, β1‐integrin returns to the cell membrane through the intracellular long circulation pathway to complete the recycling process. In turn, more β1‐integrin activates the FAK/ERK pathway to promote the progression of NSCLC. In this process, both TBC1D23 and RAB11A are indispensable.

Moreover, β1 integrin was also present in our IP‐MS results (Figure S2B), suggesting that TBC1D23 may also interact with β1 integrin. If this is the case, TBC1D23 most likely recruits β1 integrin to the RAB11A‐mediated PERC, connecting RAB11A to β1 integrin and thus mediating circulation of β1 integrin. The decreased amount of β1 integrin that eventually returns to the cell membrane is due to decreased β1 integrin content in the PERC. Therefore, we plan to use isotopes to label TBC1D23, RAB11A and β1‐integrin in further experimental studies to observe their real‐time intracellular localization.

Our study had some limitations. We did not construct a mutation in TBC1D23 nor detect which fragment of TBC1D23 interacted with RAB11A. These provide avenues for future studies. And our study provides insight into the relationship between TBC1D23 expression and adverse clinicopathological characteristics of patients with NSCLC, suggesting that TBC1D23 is an important target for treatment.

## CONFLICT OF INTEREST

The authors declare that they have no conflict of interests.

## AUTHOR CONTRIBUTION

Yao Zhang: Conceptualization (lead); Data curation (lead); Formal analysis (lead); Methodology (lead); Project administration (equal); Resources (lead); Software (lead); Writing‐original draft (lead); Writing‐review & editing (lead). Hongbo Su: Conceptualization (supporting). Muli Wudu: Data curation (supporting). Hongjiu Ren: Formal analysis (supporting). Yitong Xu: Methodology (supporting). Qingfu Zhang: Software (supporting). Jun Jiang: Data curation (supporting). Qiongzi Wang: Data curation (supporting). Xizi Jiang: Software (supporting). Bo Zhang: Software (supporting). Zongang Liu: Software (supporting). Zifang Zou: Formal analysis (supporting). Xueshan Qiu: Funding acquisition (lead); Project administration (lead).

## Supporting information

Fig S1

Fig S2

Fig S3

Table S1

## Data Availability

All data generated or analysed during this study are included in this published article. Further data are available from the corresponding author upon request.
