# Supplementary figures and images for: TBC1 domain family member 23 interacts with Ras‐related protein Rab‐11A to promote poor prognosis of non‐small‐cell lung cancer via β1‐integrin
Source: J Cell Mol Med. 2021 Aug 7;25(18):8821–35. doi: 10.1111/jcmm.16841 (PMC8435452; doi:10.1111/jcmm.16841)

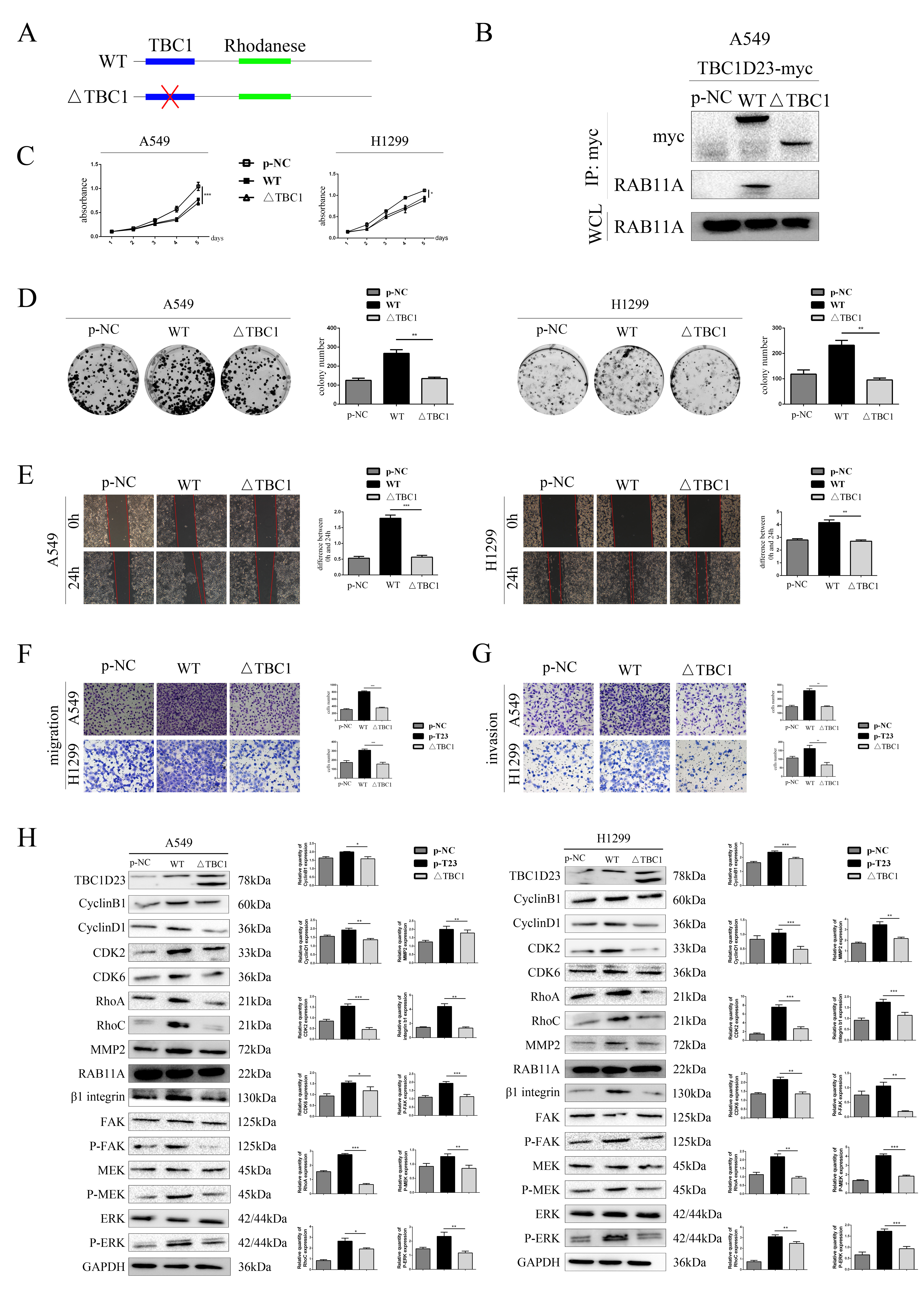

Supplement: Supplementary file 1 — Fig S1 [file JCMM-25-8821-s001.jpg]

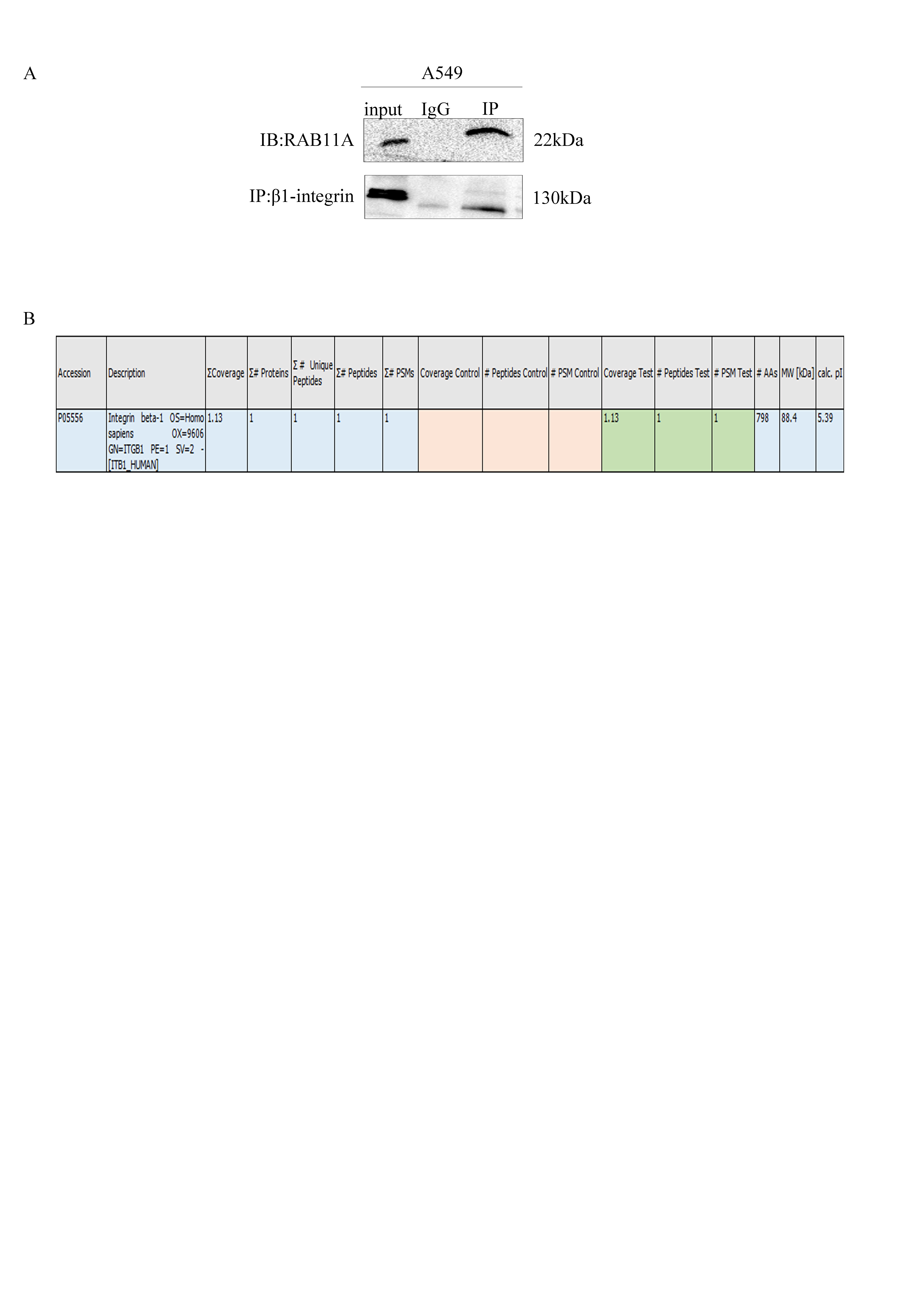

Supplement: Supplementary file 2 — Fig S2 [file JCMM-25-8821-s002.jpg]

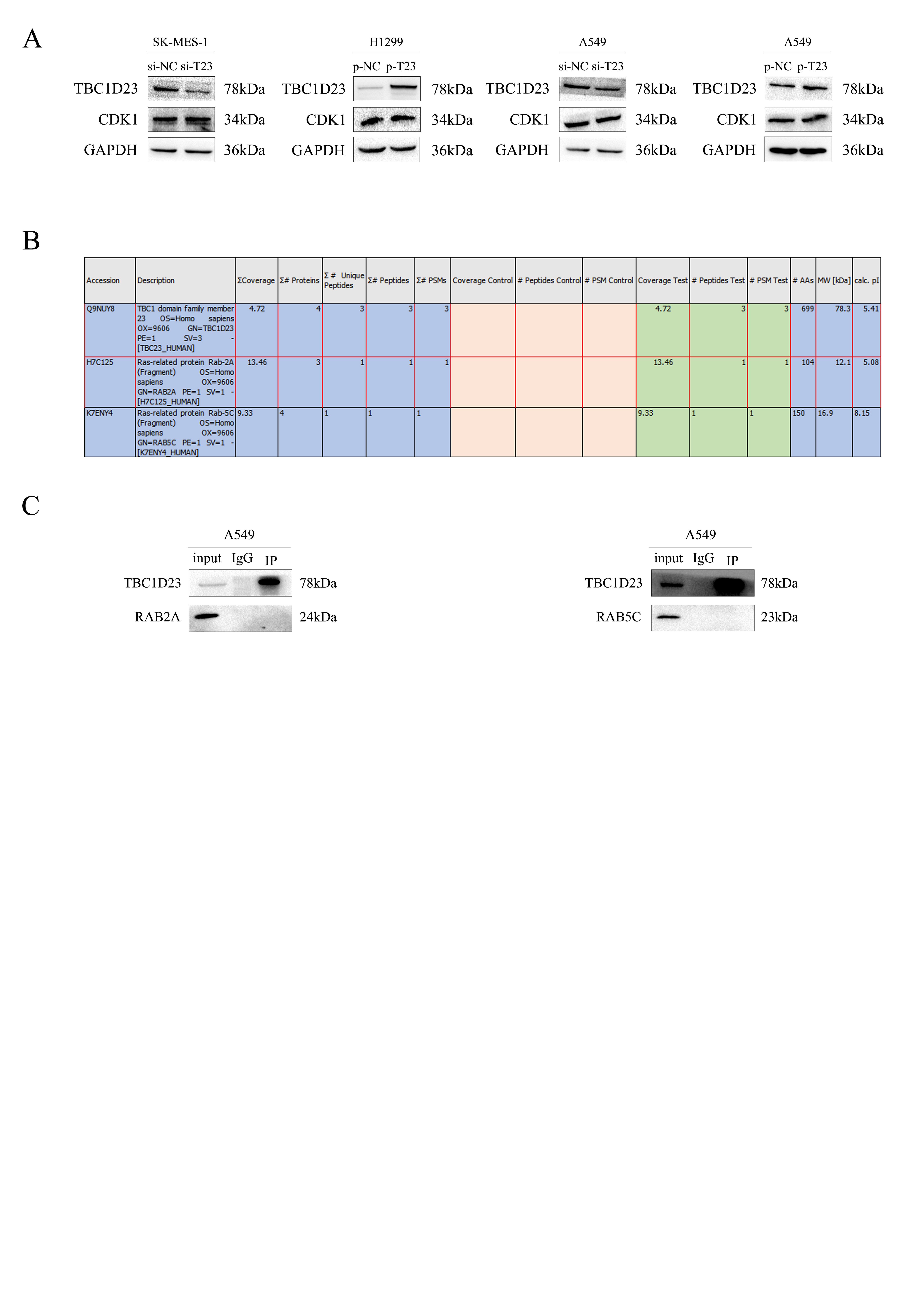

Supplement: Supplementary file 3 — Fig S3 [file JCMM-25-8821-s003.jpg]
